# Abbreviated MDS-UPDRS for Remote Monitoring in PD Identified Using Exhaustive Computational Search

**DOI:** 10.1155/2022/2920255

**Published:** 2022-06-07

**Authors:** Gareth Morinan, Robert A. Hauser, Anette Schrag, Jingxuan Tang, Jonathan O'Keeffe, MDS-NMS Scale Development Study Group

**Affiliations:** ^1^Machine Medicine Technologies Ltd., The Leather Market Unit 1.1.1, 11/13 Weston Street, London SE1 3ER, UK; ^2^Parkinson's Disease and Movement Disorders Center, Department of Neurology, Parkinson Foundation Center of Excellence, University of South Florida, 4001 E Fletcher Ave, Tampa, FL 33613, USA; ^3^Department of Clinical and Movement Neurosciences, Institute of Neurology, University College London, Queen Square, London WC1N 3AR, UK; ^4^Movement Disorder Society, 555 East Wells Street, Suite 1100 Milwaukee, WI 53202-3823, USA

## Abstract

**Background:**

The Movement Disorder Society Unified Parkinson's Disease Rating Scale (MDS-UPDRS) comprises 50 items, consisting of historical questions and motor ratings, typically taking around 30 minutes to complete. We sought to identify an abbreviated version that could facilitate use in clinical practice or used remotely via telemedicine.

**Methods:**

To create an 8-item version we conducted an “exhaustive search” of all possible subsets. We measured explained variance in comparison to the 50-item version using linear regression, with the “optimal” subset maximising this while also meeting remote assessment practicality constraints. The subset was identified using a dataset collected by the Parkinson's Progression Markers Initiative and validated using an MDS Non-Motor Symptoms Scale validation study dataset.

**Results:**

The optimal remote version comprised items from all parts of the MDS-UPDRS and was found to act as an unbiased estimator of the total 50-item score. This version had an explained variance score of 0.844 and was highly correlated with the total MDS-UPDRS score (Pearson's *r* = 0.919, *p*-value <0.0001). Another subset that maximised explained variance score without adhering to remote assessment practicality constraints provided similar results.

**Conclusion:**

This result demonstrates that the total scores of an abbreviated form identified by computational statistics had high agreement with the MDS-UPDRS total score. Whilst it cannot capture the richness of information of the full MDS-UPDRS, it can be used to create a total score where practicality limits the application of the full MDS-UPDRS, such as remote monitoring. Further validation will be required, including in specific subgroups and advanced disease stages, and full validation of clinimetric properties.

## 1. Introduction

Severity of Parkinson's disease (PD) is commonly assessed using the Movement Disorder Society Unified PD Rating Scale (MDS-UPDRS), consisting of historical questions and motor ratings, with an average completion time of around 30 minutes [1]. The MDS-UPDRS consists of four parts, requiring a total of 65 ratings made on a 5-point (0–4) scale (see Supplement Table 1).

Previous work has suggested the duration of assessment required for a PD rating scale can restrict adoption in clinical practice [2–4]. Creation of a short version would reduce patient and clinician burden. It is also of increasing interest given information technology advances and rising demand for remote assessment, with shorter and potentially higher frequency assessments. The MDS-UPDRS has not yet been validated for remote use.

Even in most developed countries, public access to healthcare is still restricted [5]. Telemedicine can benefit patients by saving cost and time spent attending appointments and reducing travel [6]. Preliminary research suggested that telemedicine for PD is feasible [7, 8], and most of the PD motor examinations can be performed remotely [7, 9].

Previous work proposed an 8-item shortened version of the original UPDRS based upon clinical relevance and expertise [4]. We aimed to develop an 8-item version of the MDS-UPDRS which minimises information loss, measured using explained variance, compared to the MDS-UPDRS total score, while meeting remote assessment practicality constraints. This was achieved by conducting an “exhaustive search” (testing every possible 8-item combination).

## 2. Methods

### 2.1. Data

We utilised two datasets for this work: a “training” dataset, which was used to determine the optimal 8-item subset, and a “validation” dataset, which was used to validate the effectiveness of the optimal remote subset on a patient group that was independent of the training dataset.

For training we used the Parkinson's Progression Markers Initiative (PPMI) dataset [10] (accessed 12 July 2021), while for validation we used the MDS-UPDRS scores collected in the MDS-Non-Motor Symptoms Scale (MDS-NMS) validation study [11]. Both datasets contain MDS-UPDRS examinations conducted by trained assessors, performed across multiple sites.

For an assessment to be included in this analysis, we required it to have a rating for every MDS-UPDRS item. Of the 15,986 assessments in the training dataset, 7,594 satisfied this requirement, while of the 402 assessments in the validation dataset, 377 did. This selection process did not introduce a bias with respect to age or gender (see Supplement Table 2).

The MDS-UPDRS rating distributions for these two datasets were visually similar; however, the median total MDS-UPDRS score of the validation dataset was significantly higher (Mann–Whitney's *U* = 1153785, *p*-value <0.0001) [12]. The median Hoehn and Yahr stage [13] was the same. For a full comparison between datasets, see Supplement Figure 1 and Supplement Table 3.

### 2.2. Remote Assessment Practicality Constraints

We set out to find a subset of 8 MDS-UPDRS items that would be practical for high-frequency remote assessment, which meant excluding certain items from consideration. We excluded items that require face-to-face assessments, which are 3.3 Rigidity and 3.12 Postural Stability [1]. Also, as patients generally carry out remote assessments using an electronic device placed on a desk or table in front of them and assessment of lower body movements and observation of gait requires repositioning of the device or patient, we excluded six items that require the lower limbs of the patient to be observed (items 3.7 Toe Tapping, 3.8 Leg Agility, 3.10 Gait, 3.11 Freezing of Gait, 3.13 Posture, and 3.14 Body Bradykinesia [1]).

### 2.3. Identification of MDS-UPDRS Subsets

While our ultimate aim was to identify an “optimal” subset for remote assessment, and certain items were not be included due to practicality constraints, we began by carrying out an exhaustive search of every possible 8-item combination of the 50 MDS-UPDRS items. This required evaluating 536,878,650 (“50 choose 8”) possible subsets and was done in order to measure, using our evaluation metrics, the impact of applying remote practicality constraints. The optimal subset across all combinations was termed the “any-item” subset, and the subset that was optimal while meeting practicality constraints was termed the “remote” subset.

### 2.4. Evaluation Metrics

Our criterion for model selection was the model that maximised the primary metric, the explained variance score (EVS) [14] of a linear regression. To verify this procedure, we used two secondary metrics: Spearman's Rank Correlation Coefficient (SRC) [15] and Pearson's Correlation Coefficient (PCC) [16]. Supplement Section 2.4 provides more detail on metric computation.

## 3. Results

### 3.1. Exhaustive Search

An exhaustive search was conducted of the 536,878,650 possible 8-item combinations. The distribution of the three metrics, computed using the training dataset, can be seen in the Supplement Figure 2 and Supplement Table 4. Examining the top 100,000 ranked subsets found the rate of occurrence of items varied considerably (see Supplement Figure 3 for occurrences of each item); however, item 1.13 Fatigue appeared in more than 50% of these subsets, indicating it is relatively informative, whereas 18 different items appeared in less than 5% of these subsets, such as item 3.3 Rigidity, which was the motor examination item with the lowest rate of occurrence.

### 3.2. Highest Ranked 8-Item Subsets

Ranking all subsets by EVS, we arrived at the optimal “remote” and “any-item” subsets (see Table 1). The two subsets included similar clinical features although half of the individual items differed between them. Items 1.13 Fatigue, 2.5 Dressing, 2.10 Tremor, and 4.3 Time Spent in the Off State were included in both, whereas 3.2 Facial Movement, 3.4 Finger Tapping, 2.12 Walking and Balance, and 3.9 Arising From Chair appeared in the remote subset and 2.1 Speech, 3.5 Hand Movement, 3.7 Toe Tapping, and 3.13 Posture appeared in the any-item subset.

The statistical metrics for these two subsets were similar (remote, any-item; EVS = 0.844, 0.847; PCC = 0.919, 0.920; SRC = 0.900, 0.905, see Supplement Table 5). The correlation between the sum of the 8-item ratings for these two subsets was highly significant by both PCC (Pearson's *r* = 0.898, *p*-value <0.0001) and SRC (Spearman's *r* = 0.880, *p*-value <0.0001), see Supplement Figure 4.

The remote subset was ranked 20th among all subsets evaluated. Furthermore, we found a large overlap in the 95% credible intervals for each of the 40 highest ranked subsets (see Supplement Figure 5 and Supplement Table 6 for comparison of, and constituent items within, each of the top 40 subsets). This suggests the amount of information contained within each of these 40 subsets is similar, and so the application of remote assessment practicality constraints (i.e., selecting the 20th ranked by EVS, instead of the 1st) has minimal impact upon our evaluation metric.

### 3.3. Validation Dataset

**Figure 1:**
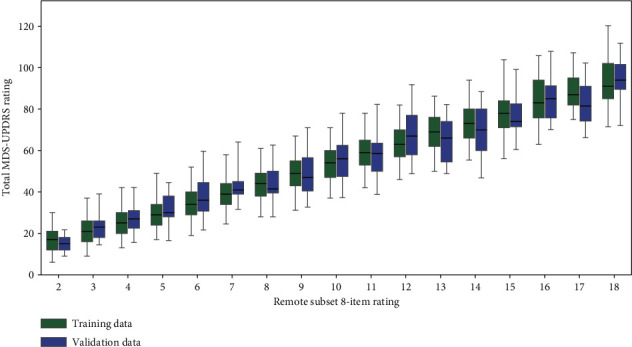
Boxplots showing the distribution of the sum rating of the total 50-item MDS-UPDRS rating for each value of the remote subset rating (see Table 1) for both training and validation datasets, with the interquartile range indicated by the boxes and the range from the 2.5th to 97.5th percentile indicated by the whiskers. Note this figure only displays boxplots of 8-item ratings for which *n* ≥ 5, e.g., 8-item rating of zero is not displayed because *n* ≥ 5 in the validation dataset (Supplement Table 8 shows the (*n*) for each rating). For each of these ratings, there is a large overlap between the range from 2.5th to 97.5th percentile of the training and validation datasets.

We tested the remote subset on the validation dataset, to examine whether the degree of explained variance was similar when applied to a different patient group. For each possible 8-item rating, the range of possible total 50-item ratings was similar in both the training and validation sets (see Figure 1).

The statistical metrics were similar between the training and validation datasets (training, validation; EVS = 0.844, 0.805; PCC = 0.919, 0.897; SRC = 0.900, 0.888, see Supplement Table 7). The 95% credible intervals of the two datasets overlapped for each metric (see Supplement Figure 6).

### 3.4. Subset Explanatory Power

**Figure 2:**
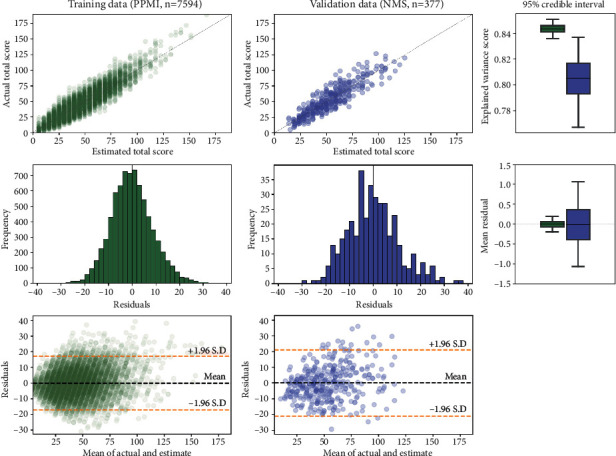
A linear regression model was used to estimate the total 50-item MDS-UPDRS rating (see Section 2.4), using the 8-item rating of the “remote” subset (see Table 1). (Upper left) The correspondence between ground truth and estimations for the training dataset, and (upper center) the validation dataset. Note: the *X* = *Y* line is marked in grey. For both datasets, the correlations between ground truth and estimations were highly significant (*p*-value <0.0001), and (upper right) the 95% credible intervals of these correlations overlapped. This suggests the ability of this 8-item subset to estimate the total 50-item MDS-UPDRS rating generalises across different datasets. (Mid left) The residuals of the estimator for the training dataset, and (mid center) the validation dataset. The mean residuals for both datasets were close to zero, and (mid right) the 95% credible intervals of the mean residual crossed zero for both datasets. This suggests the linear regression model is an unbiased estimator of the total 50-item MDS-UPDRS rating. (Lower left) The Bland–Altman plot of agreement between the actual and estimated total score for training (lower center) and validation, both of which show that residuals are approximately equally distributed either side of zero across the range of possible values of total score, indicating a lack of proportional bias.

The linear regression model (described in Section 2.4) employing the remote 8-item subset was used to estimate the total 50-item MDS-UPDRS ratings. Comparing these estimations to the 50-item rating (upper row Figure 2) showed a highly significant correlation for both the training dataset (Pearson's *r* = 0.919, *p*-value <0.0001) and the validation dataset (Pearson's *r* = 0.897, *p*-value <0.0001). The equation of the model trained on the entire training dataset was *y* = 4.87*x* + 5.59, where *x* is the sum of the subset ratings. While this equation could be used to convert between the 8-item and 50-item scales, the resulting 50-item rating would only be an approximation (as seen in Figure 1, there was a range of 50-item ratings for each possible 8-item rating) rather than an equivalent score.

Use of the remote 8-item subset to estimate the 50-item rating provided an unbiased estimator, as can be seen from the credible interval of the mean residuals of this linear model estimator being centered on zero (lower row Figure 2). We note there is a slight right skew in the residuals of the training dataset, which can also be observed in the scatter-plot (upper left Figure 2) where it can be seen that the model tends to under-estimate when the actual MDS-UPDRS rating is above 100. This means that, while the estimator is unbiased on average, there is a conditional bias for patients with very high MDS-UPDRS ratings, and therefore care should be taken in interpreting results in such cases.

## 4. Discussion

### 4.1. Summary of Result

We aimed to provide an abbreviated 8-item version of the 50-item MDS-UPDRS that was suitable for remote assessment through identifying a subset of items that minimised information loss, as measured by explained variance with respect to the MDS-UPDRS total score, while adhering to practicality constraints. We conducted an exhaustive search of all possible 8-item subsets of the 50-items (*n* = 536,878,650). This computationally intensive approach resulted in finding subsets for which the sum ratings were highly correlated (Pearson's *r* > 0.9) with the full 50-item UPDRS.

The subset that maximised explained variance, while adhering to the remote assessment practicality constraints, contained three motor examination items (3.2 Facial Movement, 3.4 Finger Tapping, 3.9 Arising From Chair) along with five question items (1.13 Fatigue, 2.5 Dressing, 2.10 Tremor, 2.12 Walking and Balance, 4.3 Time Spent in the Off State).

We found the explained variance for this “remote” subset (EVS = 0.844) and the “any-item” subset (EVS = 0.847) were similar (their 95% credible intervals overlapped), and these subsets shared four of the same items.

We demonstrated that our results are robust by testing this remote subset, selected using the training dataset, on a separate validation dataset. The explained variance on this validation dataset was found to be slightly lower (EVS = 0.805); however, the 95% credible intervals of explained variance of the two datasets overlapped.

Finally, we show that the linear regression model constructed using this remote subset acts as an unbiased estimator of the total 50-item rating, as seen from the 95% credible interval of the mean residual of this model being centered on zero for both training and validation datasets.

### 4.2. Comparison to Previous Work

Previous work proposed an 8-item subset of the original UPDRS based upon clinical experience [4]. Whilst this is not directly comparable as it was a subset of the original UPDRS [17], approximately mapping these 8 items from the original UPDRS to items from the MDS-UPDRS, we find this previous subset has lower explained variance (EVS = 0.681) than the ones identified by our analyses. However, this is unsurprising as the methodology and motivation behind the previous work was not statistical optimisation of explained variance with respect to the total score, but selection of items based on the individual item clinical relevance. For example, item 1.3 Depressed Mood, which was included in the previous work's 8-item scale is a recognised important clinical feature in that if a patient is depressed he may benefit from antidepressant treatment. However, our work found it to be relatively uninformative for estimating the total score. Similarly, item 4.1 Time Spent with Dyskinesias was uninformative for estimation of the total score, most likely because dyskinesias affect a relatively small proportion of patients. Our proposed 8-item subset is therefore intended to provide information on the overall disease severity equivalent to the total MDS-UPDRS score. Our proposal does not aim to provide the same comprehensive clinical information regarding individual items of the MDS-UPDRS or individual aspects of PD that might require treatment. Rather, similar to the total MDS-UPDRS score, it can be considered an index of overall disease severity.

### 4.3. Limitations and Future Work

The selection of our proposed 8-item subset relied upon the training dataset, which primarily represents patients in earlier stages of PD. Patients included in the validation dataset had greater later stage representation, but the results were similar, indicating the results are robust. Nevertheless, future work should examine whether other 8-item subsets may be more appropriate for later disease stages.

The use of an 8-item scale will lead to measurements that have a greater level of noise compared to 50-items, particularly for later stage PD. This means that comparisons between patients, particularly those with later stage PD, using the 8-item rating should be done with caution. We believe the scale will have more practical benefit for intrapatient comparison, comparing a patient over time, rather than interpatient comparison.

We note that the nature of our statistical approach means we inevitably arrived at a subset that was optimised for the “average” PD patient. Other approaches, such as the previous work discussed above, could result in a subset that may be more useful to specific patients and patient groups but slightly less so for the majority. Future work could examine ways of segmenting patient populations and the intent of assessments in order to arrive at differently optimised subsets for different patient groups. For instance, one subset might be more useful for patients in a research setting vs clinical care or be better suited for patients who are being considered for deep brain stimulation.

Our method quantifies information loss with respect to the total MDS-UPDRS score, but for certain monitoring purposes only the motor examination (part III) may be relevant, and for others only the aspects of daily living (parts I & II). We note the 8-item subset resulting from our approach consists mostly of motor items (parts II & III) and includes only a single part I nonmotor item, meaning there is greater information loss with respect to nonmotor symptoms as compared to motor symptoms. Future work could identify item subsets that maximise different parts of the MDS-UPDRS, such as a 5-item nonmotor assessment.

The MDS-UPDRS has not yet been validated for remote use. If and when this validation occurs, future work could validate our abbreviated scale by a similar method.

Lastly, our proposed 8-item subset is not suitable for use as a diagnostic tool, just as the full MDS-UPDRS is not a diagnostic tool. We note in particular that our 8-item subset does not include rigidity, which the PD diagnosis criteria mention as a key feature of the disease. Thus, our 8-item subset should only be administered in patients who have received a PD diagnosis. Additionally, the 8-item scale is not directly comparable to, and therefore should not replace, the full MDS-UPDRS scale.

### 4.4. Practical Application

We estimate that for the vast majority of PD patients the “remote” 8-item assessments could be completed in 5–10 minutes. This estimate is based upon each motor examination item typically taking 30–60 seconds to complete, along with an assumption that each question would take 15–30 seconds, and 2-3 minutes being required to initially set up the assessment and transition between items.

Outside of clinical trials and academic centers, MDS-UPDRS ratings are rarely performed. This appears to be due to the time required, which is estimated to be about 30 minutes. In addition, many PD clinical visits are being performed via telehealth, and we suspect even fewer MDS-UPDRS ratings are performed under these circumstances due to additional barriers, including difficulty visualizing various body parts and performance tasks. We have therefore derived brief, 8-item versions of the MDS-UPDRS that correlate well with the overall total MDS-UPDRS score. These scales, an any-item scale that can be used in person and a remote scale that can be used for telehealth visits, can be used as an overall index of disease status and only add an additional 5–10 minutes. We believe this may be appealing to busy clinicians as an adjunct to their usual history and exam.

Our result demonstrates that an 8-item subset identified by computational statistics appears to be a robust abbreviated version of the total MDS-UPDRS, which could be used to create a total score where practicality limits the application of the full MDS-UPDRS. Additionally, given this subset was constrained to not include items that may be impractical for home assessment, this version is well suited for remote monitoring.

## Figures and Tables

**Table 1:** Items contained within the subsets that rank highest, by explained variance score on the training dataset, while adhering (“remote”) or not adhering (“any-item”) to the remote assessment constraints defined in Section 2.3. These subsets share four of the same items, and both correspond to the same core aspects of Parkinson's disease; movement of upper limbs (3.4 or 3.5) and lower limbs (3.9 or 3.7), tremor (2.10), axial (2.12 or 3.13), speech/facial muscles (3.2 or 2.1), activities of daily living (2.5), fatigue (1.13), and time in Off (4.3).

Remote subset	Any-item subset
(1.13) fatigue	(1.13) fatigue
(2.5) dressing	(2.1) speech
(2.10) tremor	(2.5) dressing
(2.12) walking and balance	(2.10) tremor
(3.2) facial movement	(3.5) hand movement
(3.4) finger tapping	(3.7) toe tapping
(3.9) arising from chair	(3.13) posture
(4.3) time spent in the off state	(4.3) time spent in the off state

## Data Availability

The Parkinson's disease assessment data used to support the findings of this study were supplied by the Parkinson's Progression Markers Initiative (PPMI). Qualified researchers may obtain access by direct request to the PPMI (https://www.ppmi-info.org/). Additional Parkinson's disease assessment data used to support the findings of this study were supplied by the Movement Disorder Society (MDS) Non-Motor Symptoms Scale Development Study Group. Requests for access to these data should be made to the MDS (https://www.movementdisorders.org/).

## Supplementary Materials

Supplementary Materials The supplementary materials report included with this paper contains additional tables and figures, as well as a more detailed explanation of the statistical methodology.
