# Supplementary material for: Abbreviated MDS-UPDRS for Remote Monitoring in PD Identified Using Exhaustive Computational Search
Source: Parkinsons Dis. 2022 Jun 7;2022:2920255. doi: 10.1155/2022/2920255 (PMC9197633; doi:10.1155/2022/2920255)
Supplement: Supplementary Materials — The supplementary materials report included with this paper contains additional tables and figures, as well as a more detailed explanation of the statistical methodology. [file 2920255.f1.docx]

Abbreviated MDS-UPDRS for remote monitoring in

PD identified using exhaustive computational search: supplementary materials

**1. Introduction**

# 1.1. Overview of MDS-UPDRS

| **Part** | **UPDRS Assessment** | **Items** | **Ratings** |
| --- | --- | --- | --- |
| I | Non-Motor Aspects of Experiences of Daily  Living (nM-EDL) | 13 | 13 |
| II | Motor Aspects of Experiences of Daily Living  (M-EDL) | 13 | 13 |
| III | Motor Examination | 18 | 33 |
| IV | Motor Complications | 6 | 6 |

Table 1: Summary of the number of items and ratings required for each part of the MDSUPDRS [1] examination.

**2. Methods**

# 2.1. Excluded data

| **Measure** |  | **Entire PPMI** | **Training** |
| --- | --- | --- | --- |
| Number of assessments |  | 15986 | 7594 |
| Percentage female |  | 58.3 | 60.6 |
| Age | Lower quartile  Median | 55  62 | 54  62 |
|  | Upper quartile | 68 | 68 |

Table 2: Comparison between the full PPMI dataset and the training data, which consists only of PPMI assessment for which every rating was recorded. The distribution of age and gender is similar between the two, which indicates that excluding incomplete assessments did not introduce a selection bias with respect to gender or age.

# 2.2. Data overview

| **Measure** |  | **Training** | **Validation** |
| --- | --- | --- | --- |
| Number | Assessments  Patients | 7594  913 | 377  377 |
|  | Sites | 33 | 6 |
| Total MDS-UPDRS score | Lower quartile  Median | 29  42 | 36  50 |
|  | Upper quartile | 57 | 68 |
|  | Standard deviation | 22.1 | 24.5 |
| Hoehn & Yahr | Median | 2 | 2 |
|  | Standard deviation | 0.64 | 0.75 |

Table 3: Summary of training and validation datasets. These figures concern only the subset of each dataset used for our analysis. The total MDS-UPDRS score refers to the sum of ratings in MDS-UPDRS Parts I-IV.

# 2.3. Data distributions


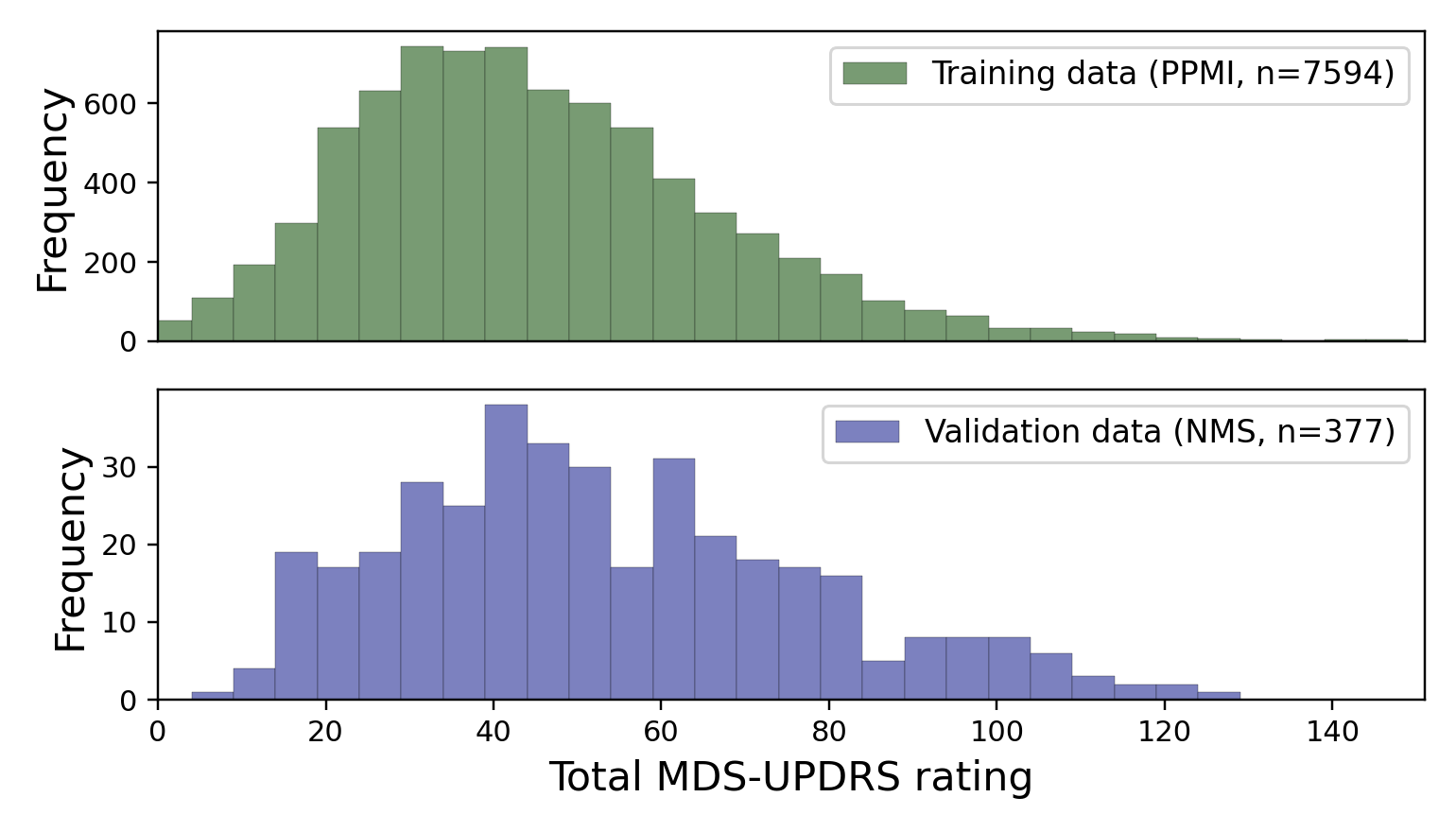


Figure 1: Distribution of total MDS-UPDRS score (sum of all ratings for parts I, II, III and IV) within the training and validation datasets.

# 2.4. Evaluation Metrics & Regression Model

For each subset we evaluated information loss using three metrics. We used bootstrapping [2] to generate 95% credible intervals of these metrics to compare different results. (Interval constructed using 10,000 bootstrap iterations, each iteration sampling with replacement from the relevant dataset; n = 7594 for training, n = 377 for validation).

Our primary evaluation metric was the explained variance score (EVS) [3] of a linear regression model, that used a single input feature which was the 8-item score (i.e. the sum of all ratings associated with those 8-items), to estimate the 50 item score (i.e. the sum of all 65 ratings). Estimations on the training dataset were made using 5-fold cross validation [4], while for the validation dataset estimates were made using a model trained on the entire training dataset. We used bootstrapping to generate 95% credible intervals for the mean residual of this model, and consider the model unbiased if the credible intervals for both training and validation datasets crossed zero.

Our secondary evaluation metrics were the Spearman’s Rank Correlation Coefficient (SRC) [5] and Pearson’s Correlation Coefficient (PCC) [6] between the 8-item and the 50-item scores.

Our criterion for model selection was the model that maximised our primary evaluation metric, with the secondary evaluation metrics being used to confirm this selection.

**3. Results**

# 3.1. Exhaustive search overview


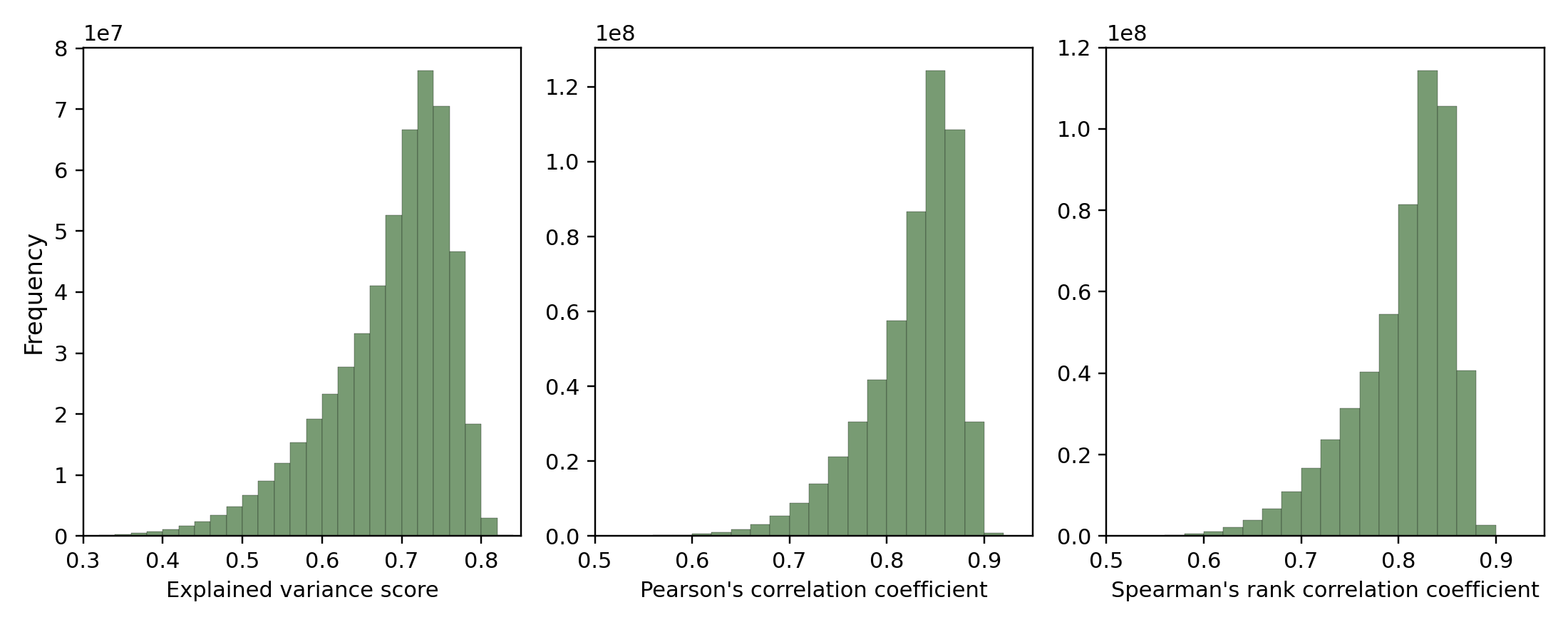


Figure 2: Histograms showing the distributions of each metric for each of the 8-item subsets (n = 536,878,650), computed on the training dataset. For the explained variance score the modal bin was 0.72 - 0.74. For the Pearson correlation coefficient the modal bin was 0.84 - 0.86. For the Spearman rank correlation coefficient the modal bin was 0.82 - 0.84. Each metric shows a similarly shaped distribution, suggesting that all three metrics are measuring information loss in a similar fashion.

|  | Minimum bin | Modal bin | Maximum bin |
| --- | --- | --- | --- |
| EVS | 0.16 - 0.18 | 0.72 - 0.74 | 0.84 - 0.86 |
| PCC | 0.40 - 0.42 | 0.84 - 0.86 | 0.92 - 0.94 |
| SRC | 0.40 - 0.42 | 0.82 - 0.84 | 0.90 - 0.92 |

Table 4: Summary of histograms bins of metrics (see Figure 2) computed during the exhaustive search of all 8-item subsets, computed on the training dataset. (EVS = explained variance score, PCC = Pearson’s correlation coefficient, SRC = Spearman’s rank correlation coefficient).


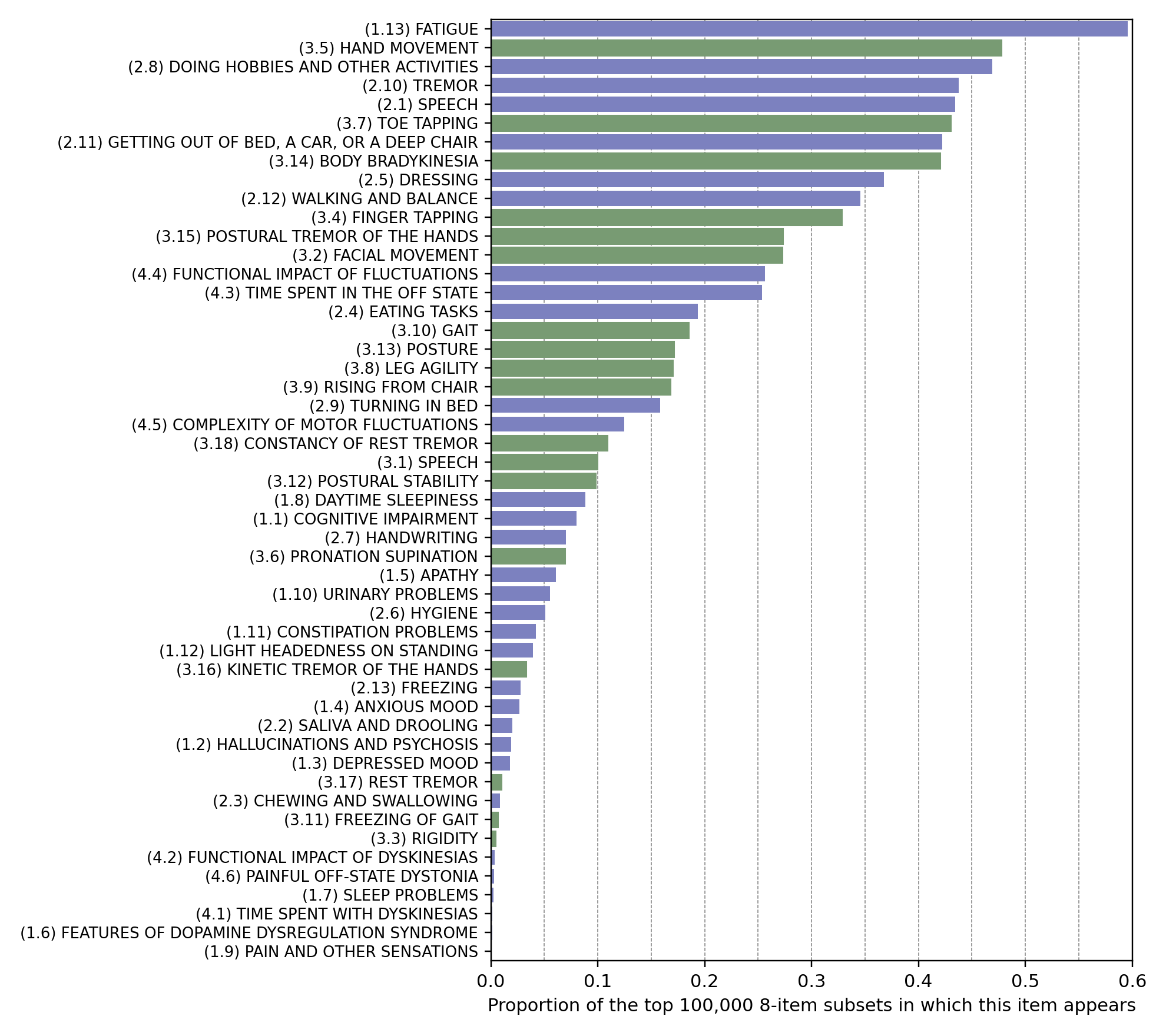


Figure 3: How often each item appears within the top 100,000 8-item subsets, as ranked by explained variance score computed on the training dataset. Motor examination items are coloured green, while question items are coloured blue. Only one item 1.13 Fatigue appears in more than 50% of these subsets. 18 items appear in less than 5% of subsets, such as item 3.3 Rigidity, which is the motor examination item with the least occurrences.

# 3.2. Any-item and remote subsets

|  | Any-item subset | Remote subset |
| --- | --- | --- |
| EVS | 0.847 | 0.844 |
| PCC | 0.920 | 0.919 |
| SRC | 0.905 | 0.900 |

Table 5: Statistical metrics, computed on the training dataset, for the subset with the highest EVS (the “any-item” subset) as well as the subset with the highest EVS while not including any items difficult to examine remotely (the “remote” subset). The similarity of these metrics indicates that the adherence to additional practicality constraints resulted in only a very slight loss of information. (EVS = explained variance score, PCC = Pearson’s correlation coefficient, SRC = Spearman’s rank correlation coefficient).


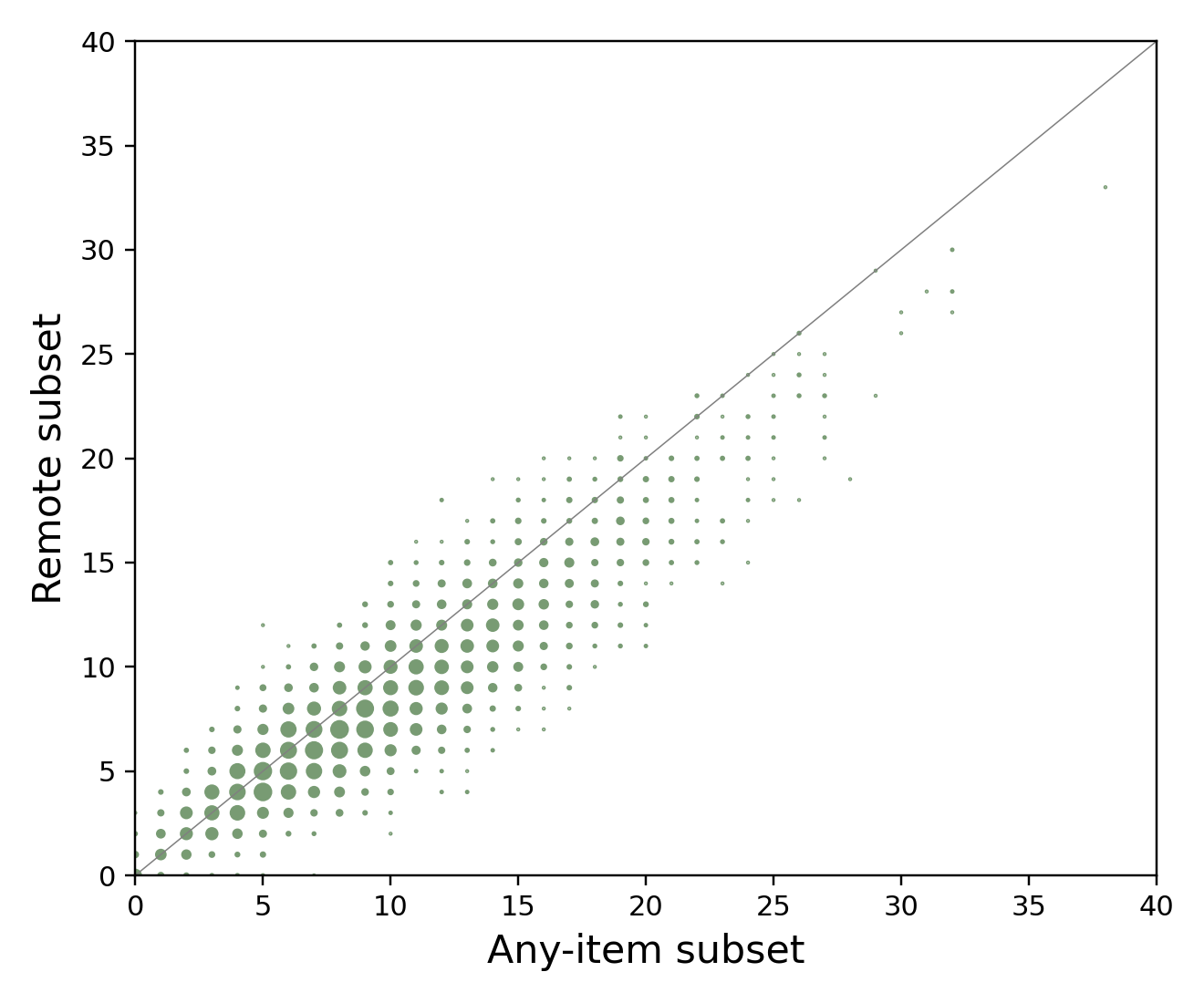


Figure 4: Correspondence between the sum rating for the any-item subset and remote subset, on the training dataset (n = 7594), where larger dots indicate more data points with those ratings, with the X = Y line marked in grey. The highly significant correlation (Pearson’s r = 0.898, p-value *<* 0.0001) suggests these subsets can be used interchangeably.

# 3.3. Top 40 subsets


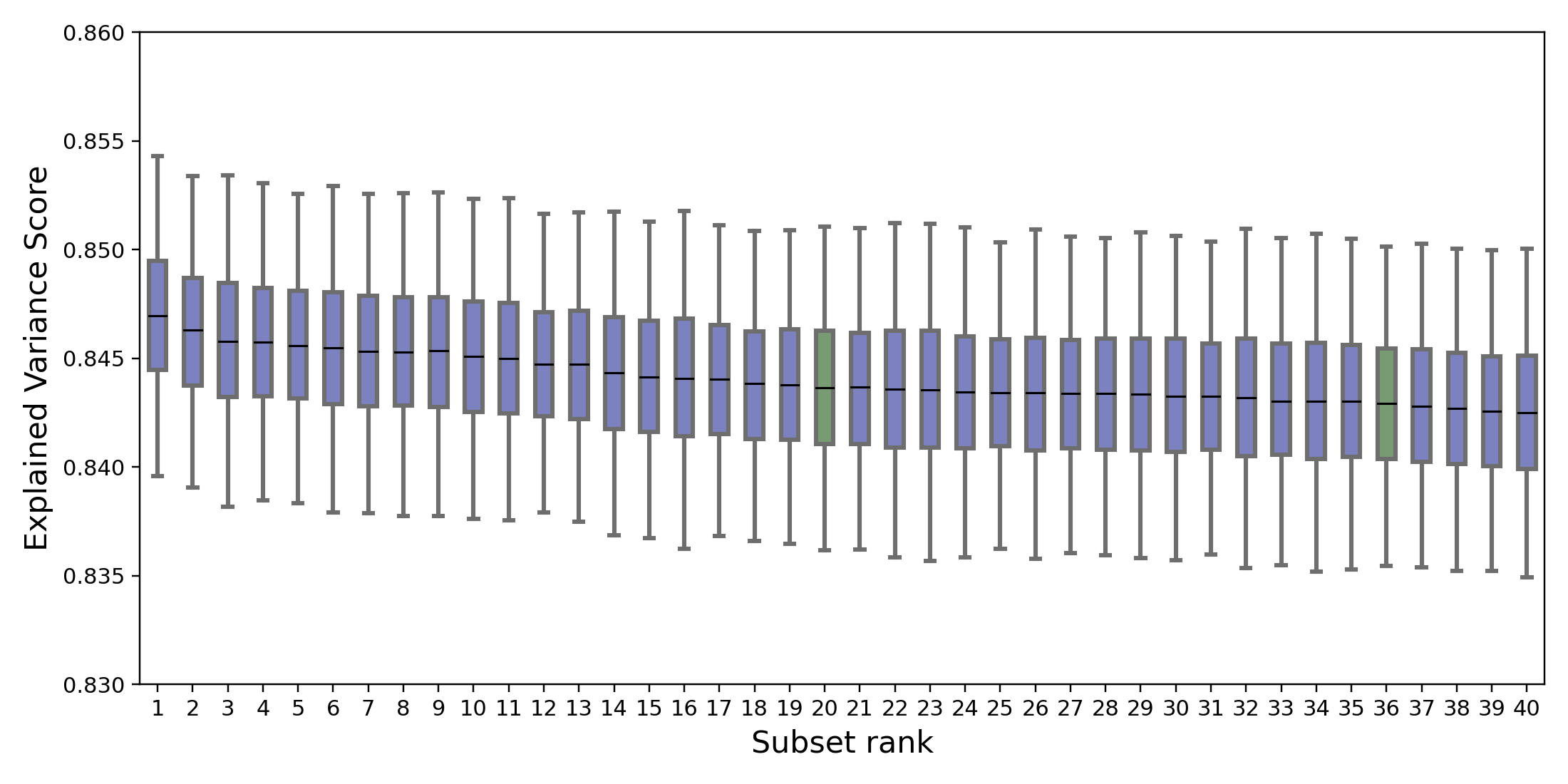


Figure 5: Bootstrap distributions of the explained variance score for each of the 40 8-item subsets that ranked highest by explained variance score, with the interquartile range indicated by the boxes and the 95% credible intervals indicated by the whiskers. Each bootstrap distribution was constructed using 10,000 iterations, each iteration computing an estimate of explained variance score by sampling with replacement from the training dataset (n = 7594). Subsets containing only remote suitable items are coloured green, other subsets are coloured blue. There is a large overlap in the 95% credible intervals for all 40 subsets, which indicates there is little difference in explained variance between any of these subsets.

| Rank |  |  |  | 8-item subset | |  |  |  | EVS | PCC | SRC | Remote |
| --- | --- | --- | --- | --- | --- | --- | --- | --- | --- | --- | --- | --- |
| 1 | 1.13 | 2.1 | 2.5 | 2.10 | 3.5 | 3.7 | 3.13 | 4.3 | .847 | .920 | .905 | False |
| 2 | 1.13 | 2.1 | 2.5 | 2.12 | 3.5 | 3.14 | 3.15 | 4.4 | .846 | .920 | .906 | False |
| 3 | 1.13 | 2.1 | 2.5 | 2.10 | 2.11 | 3.5 | 3.7 | 3.13 | .846 | .920 | .903 | False |
| 4 | 1.13 | 2.1 | 2.10 | 2.11 | 3.5 | 3.7 | 3.13 | 4.3 | .846 | .920 | .904 | False |
| 5 | 1.13 | 2.1 | 2.5 | 2.12 | 3.5 | 3.14 | 3.15 | 4.3 | .846 | .920 | .905 | False |
| 6 | 1.13 | 2.1 | 2.5 | 2.10 | 3.5 | 3.7 | 3.10 | 4.3 | .846 | .920 | .903 | False |
| 7 | 1.13 | 2.1 | 2.5 | 3.5 | 3.9 | 3.14 | 3.15 | 4.4 | .845 | .919 | .902 | False |
| 8 | 1.13 | 2.1 | 2.5 | 2.10 | 3.5 | 3.9 | 3.14 | 4.4 | .845 | .919 | .902 | False |
| 9 | 1.13 | 2.5 | 2.10 | 2.12 | 3.2 | 3.5 | 3.7 | 4.3 | .845 | .919 | .902 | False |
| 10 | 1.13 | 2.1 | 2.5 | 2.10 | 2.12 | 3.5 | 3.7 | 3.14 | .845 | .919 | .905 | False |
| 11 | 1.13 | 2.1 | 2.5 | 2.10 | 3.5 | 3.7 | 3.13 | 4.4 | .845 | .919 | .904 | False |
| 12 | 1.13 | 2.1 | 2.5 | 2.12 | 3.5 | 3.14 | 3.18 | 4.4 | .845 | .919 | .909 | False |
| 13 | 1.13 | 2.1 | 2.11 | 2.12 | 3.5 | 3.14 | 3.15 | 4.4 | .845 | .919 | .904 | False |
| 14 | 1.13 | 2.1 | 2.10 | 2.11 | 3.5 | 3.7 | 3.13 | 4.4 | .844 | .919 | .902 | False |
| 15 | 1.13 | 2.1 | 2.11 | 3.5 | 3.9 | 3.14 | 3.15 | 4.4 | .844 | .919 | .900 | False |
| 16 | 1.13 | 2.1 | 2.4 | 2.10 | 2.11 | 3.5 | 3.7 | 3.13 | .844 | .919 | .902 | False |
| 17 | 1.13 | 2.1 | 2.5 | 2.11 | 3.5 | 3.10 | 3.18 | 4.4 | .844 | .919 | .903 | False |
| 18 | 1.13 | 2.1 | 2.5 | 2.11 | 3.5 | 3.14 | 3.15 | 4.4 | .844 | .919 | .905 | False |
| 19 | 1.13 | 2.1 | 2.8 | 2.11 | 3.5 | 3.7 | 3.14 | 3.15 | .844 | .919 | .906 | False |
| 20 | 1.13 | 2.5 | 2.10 | 2.12 | 3.2 | 3.4 | 3.9 | 4.3 | .844 | .919 | .900 | True |
| 21 | 1.13 | 2.5 | 2.10 | 2.11 | 3.2 | 3.5 | 3.7 | 4.3 | .844 | .919 | .902 | False |
| 22 | 1.13 | 2.1 | 2.8 | 2.10 | 2.11 | 3.5 | 3.7 | 3.13 | .844 | .919 | .902 | False |
| 23 | 1.13 | 2.1 | 2.5 | 2.10 | 2.12 | 3.5 | 3.7 | 3.13 | .844 | .919 | .901 | False |
| 24 | 1.13 | 2.1 | 2.10 | 2.11 | 3.5 | 3.7 | 3.10 | 4.3 | .844 | .918 | .901 | False |
| 25 | 1.13 | 2.1 | 2.11 | 2.12 | 3.5 | 3.14 | 3.15 | 4.3 | .843 | .918 | .904 | False |
| 26 | 1.13 | 2.1 | 2.5 | 2.10 | 3.5 | 3.7 | 3.10 | 4.4 | .843 | .918 | .902 | False |
| 27 | 1.13 | 2.1 | 2.10 | 2.11 | 3.5 | 3.7 | 3.14 | 4.4 | .843 | .918 | .906 | False |
| 28 | 1.13 | 2.8 | 2.10 | 2.11 | 3.2 | 3.5 | 3.7 | 4.3 | .843 | .918 | .903 | False |
| 29 | 1.13 | 2.1 | 2.10 | 2.11 | 2.12 | 3.5 | 3.7 | 3.14 | .843 | .918 | .903 | False |
| 30 | 1.13 | 2.1 | 2.8 | 2.11 | 3.5 | 3.8 | 3.14 | 3.15 | .843 | .918 | .904 | False |
| 31 | 1.13 | 2.1 | 2.10 | 2.11 | 3.5 | 3.7 | 3.14 | 4.3 | .843 | .918 | .906 | False |
| 32 | 1.13 | 2.1 | 2.5 | 2.10 | 2.11 | 3.5 | 3.7 | 3.10 | .843 | .918 | .901 | False |
| 33 | 1.13 | 2.5 | 2.10 | 2.12 | 3.1 | 3.5 | 3.14 | 4.3 | .843 | .918 | .902 | False |
| 34 | 1.13 | 2.1 | 2.5 | 2.10 | 3.5 | 3.7 | 3.9 | 4.3 | .843 | .918 | .898 | False |
| 35 | 1.13 | 2.1 | 2.5 | 2.10 | 2.12 | 3.2 | 3.5 | 3.7 | .843 | .918 | .902 | False |
| 36 | 1.13 | 2.5 | 2.10 | 2.12 | 3.2 | 3.5 | 3.9 | 4.3 | .843 | .918 | .896 | True |
| 37 | 1.13 | 2.1 | 2.5 | 3.4 | 3.9 | 3.10 | 3.18 | 4.4 | .843 | .918 | .900 | False |
| 38 | 1.13 | 2.1 | 2.5 | 2.12 | 3.5 | 3.14 | 3.15 | 4.5 | .843 | .918 | .904 | False |
| 39 | 1.13 | 2.1 | 2.5 | 2.10 | 3.7 | 3.9 | 3.14 | 4.3 | .843 | .918 | .900 | False |
| 40 | 1.13 | 2.1 | 2.5 | 2.10 | 3.5 | 3.9 | 3.14 | 4.3 | .843 | .918 | .902 | False |

Table 6: The constituent items within each of the top 40 8-subsets, as ranked by EVS (explained variance score) computed on the training dataset. For the item name corresponding to each item code see Figure 3. Item 1.13 fatigue is the only item that appears in all of these subsets. The metrics values for these subsets demonstrate a high degree of agreement, indicating that the conclusion of our analysis is not that sensitive to the choice of metric.

# 3.4. Training and validation comparison

|  | Training | Validation |
| --- | --- | --- |
| Data points | 7594 | 377 |
| EVS | 0.844 | 0.805 |
| PCC | 0.919 | 0.897 |
| SRC | 0.900 | 0.888 |

Table 7: Comparison of statistical metrics, comparing the 8-item rating of the remote subset to the total 50-item rating, between training and validation datasets. The similarity of these metrics indicates that the information retention of the 8-item subset, selected using the training set, generalised to the validation dataset. (EVS = explained variance score, PCC = Pearson’s correlation coefficient, SRC = Spearman’s rank correlation coefficient).

.


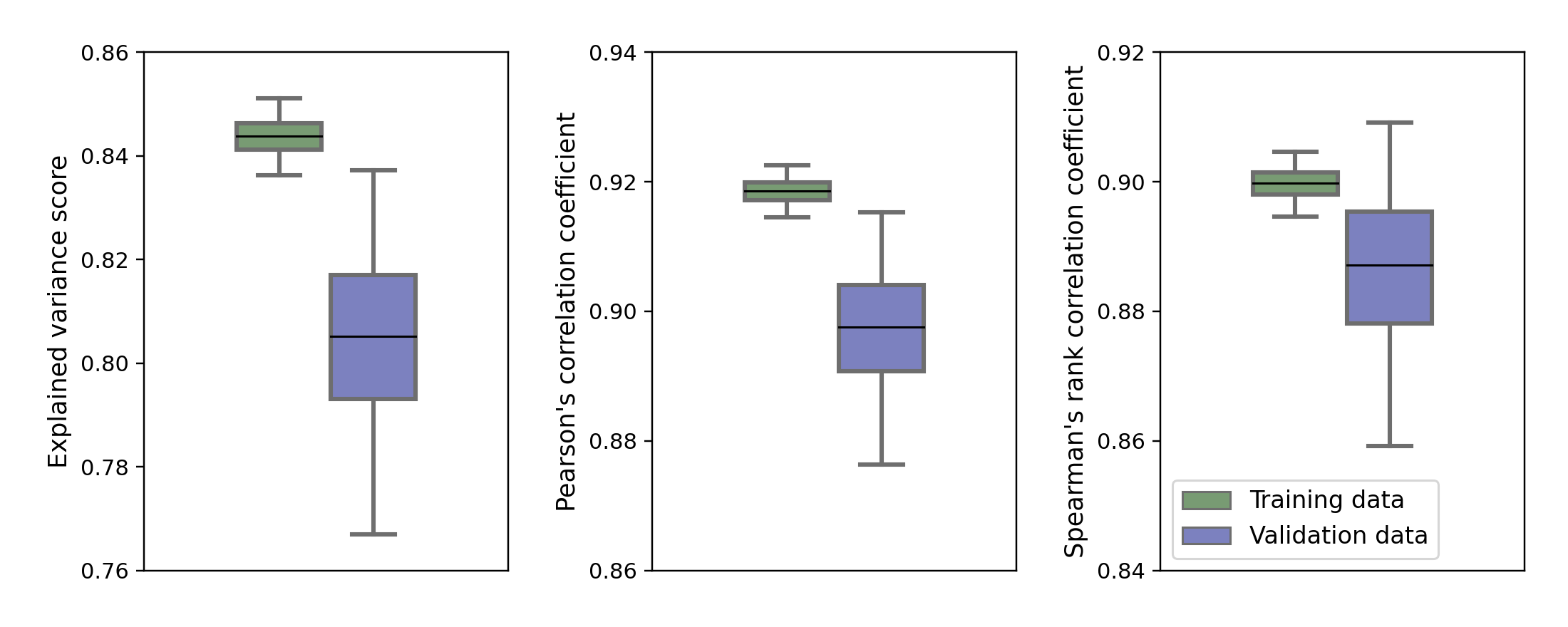


Figure 6: Box and whisker plots showing the distribution of metrics, computed through bootstrapping, for both training and validation datasets. Each bootstrap distribution was constructed using 10,000 iterations, each iteration sampling with replacement the datasets (n = 7594 for training, n = 377 for validation). The 95% credible intervals, as indicated by the whiskers, cross over considerably, indicating considerable similarity between the two datasets in terms of the information provided by this subset.

|  | Count of assessments | |
| --- | --- | --- |
| Remote subset rating | Training data | Validation data |
| 0 | 100 | 0 |
| 1 | 125 | 4 |
| 2 | 239 | 13 |
| 3 | 408 | 20 |
| 4 | 637 | 23 |
| 5 | 692 | 21 |
| 6 | 780 | 28 |
| 7 | 822 | 26 |
| 8 | 694 | 28 |
| 9 | 648 | 34 |
| 10 | 566 | 27 |
| 11 | 472 | 34 |
| 12 | 367 | 25 |
| 13 | 277 | 19 |
| 14 | 209 | 17 |
| 15 | 164 | 7 |
| 16 | 124 | 8 |
| 17 | 79 | 12 |
| 18 | 56 | 8 |
| 19 | 40 | 4 |
| 20 | 33 | 3 |
| 21 | 11 | 6 |
| 22 | 15 | 5 |
| 23 | 14 | 1 |
| 24 | 6 | 2 |
| 25 | 3 | 1 |
| 26 | 4 | 1 |
| 27 | 2 | 0 |
| 28 | 3 | 0 |
| 29 | 1 | 0 |
| 30 | 2 | 0 |
| 31 | 0 | 0 |
| 32 | 0 | 0 |
| 33 | 1 | 0 |

Table 8: Count of number of assessments for each possible value of the remote subset rating. For the validation data, many of the possible ratings have *<* 5 assessments, which is considered too few to visualise a distribution. Therefore, in Figure 2 of the main manuscript, we limit the x-axis to values 2 ≥ and ≤ 18.

**References**

1. C. G. Goetz, B. C. Tilley, S. R. Shaftman, G. T. Stebbins, S. Fahn,

P. Martinez-Martin, W. Poewe, C. Sampaio, M. B. Stern, R. Dodel, et al., Movement disorder society-sponsored revision of the unified parkinson’s disease rating scale (mds-updrs): scale presentation and clinimetric testing results, Movement disorders: official journal of the Movement Disorder Society 23 (15) (2008) 2129–2170. [doi:https://doi.org/10.1002/mds.22340.](http://dx.doi.org/https://doi.org/10.1002/mds.22340)

1. B. Efron, R. Tibshirani, An introduction to the bootstrap: Boca raton, fla (1994).
2. C. H. Achen, What does “explained variance “explain?: Reply, Political Analysis 2 (1990) 173–184.
3. M. Stone, Cross-validatory choice and assessment of statistical predictions, Journal of the Royal Statistical Society: Series B (Methodological) 36 (2) (1974) 111–133.
4. C. Spearman, Reprinted: The proof and measurement of association between two things (2010), International Journal of Epidemiology 39 (1904) 1137– 1150.
5. K. Pearson, Notes on regression and inheritance in the case of two parents proceedings of the royal society of london, 58, 240-242 (1895).
